# Acute COVID-19 is associated with altered CD8 T-cells indicative of impaired ability to control Epstein–Barr virus reactivation

**DOI:** 10.1007/s00430-026-00873-3

**Published:** 2026-04-20

**Authors:** Ulrik Stervbo, Moritz Anft, Krystallenia Paniskaki, Arturo Blazquez-Navarro, Adrian Doevelaar, Sarah Skrzypczyk, Eva Kohut, Julia Kurek, Patrizia Wehler, Sviatlana Kaliszczyk, Kamil Rosiewicz, Felix S. Seibert, Bodo Hölzer, Constantin J. Thieme, Toralf Roch, Margarethe Justine Konik, Marc Moritz Berger, Thorsten Brenner, Uwe Kölsch, Michael Adamzik, Michael Schmueck-Henneresse, Carmen Scheibenbogen, Sebastian Dolff, Ulf Dittmer, Oliver Witzke, Timm H. Westhoff, Nina Babel

**Affiliations:** 1 Center for Translational Medicine and Immune Diagnostics Laboratory, Medical Department I, Marien Hospital Herne, University Hospital of the Ruhr University Bochum, Hölkeskampring 40, 44625 Herne, Germany; 2 BIH Center for Regenerative Therapies (BCRT), Experimental Immunotherapy, Berlin Institute of Health (BIH) at Charité – Universitätsmedizin Berlin, Augustenburger Platz 1, 13353 Berlin, Germany; 3 Department of Infectious Diseases, West German Centre of Infectious Diseases, University Hospital Essen, University Duisburg-Essen, Hufelandstraße 55, 45147 Essen, Germany; 4 Charité – Universitätsmedizin Berlin, Corporate Member of Freie Universität Berlin, Humboldt-Universität Zu Berlin, Berlin Center for Advanced Therapies, Augustenburger Platz 1, 13353 Berlin, Germany; 5 Department of Anesthesiology and Intensive Care Medicine, University Hospital Essen, University Duisburg-Essen, Hufelandstraße 55, 45147 Essen, Germany; 6 Department of Immunology, Labor Berlin GmbH, Sylter Straße 2, 13353 Berlin, Germany; 7 Clinic for Anesthesiology, Intensive Care Medicine and Pain Therapy, University Hospital Knappschaftskrankenhaus Bochum, In der Schornau 23-25, 44892 Bochum, Germany; 8 Institute for Virology, University Hospital Essen, University of Duisburg-Essen, Hufelandstraße 55, 45147 Essen, Germany

**Keywords:** EBV reactivation, COVID-19, CD8 T-cells, Exhaustion

## Abstract

Increasing evidence suggests that reactivation of latent EBV in patients with COVID-19 may be linked to the development of post-acute sequelae of COVID-19, colloquially known as Long COVID. However, the reason for this co-occurrence of primary infection and reactivation of latent viruses remains elusive. During the first wave of COVID-19, we assessed all major immune cell populations by flow cytometry in a cohort of 61 patients with moderate to critical COVID-19 at the time of hospitalization. Additional blood samples from these patients were biobanked for later analysis. Using these biobanked samples, we evaluated the co-occurrence of CMV, EBV, as well as HHV-6A and -6B by qPCR. EBV was found to be reactivated not only in patients with critical or severe COVID-19 (24/33 patients; 72.72%), but also in patients with moderate COVID-19 disease (19/28; 67.86%) at the time of hospital admission. In contrast, HHV-6A was not detected among any patients, whereas CMV and HHV-6B only occurred in low frewuencies (7.1–12.1% and 10.7–15.2%, respectively). In COVID-19 patients with EBV reactivation, the degree of expression of the T-cell co-stimulatory CD28 and co-expression of CD28 and the integrin CD11a was diminished on CD8 T-cells. In contrast, the frequency of CD8 T-cells expressing the proliferative exhaustion marker CD57 increased. Collectively, these data point to an altered activation phenotype of circulating CD8 T cells and that higher replicative senescence is associated with EBV reactivation. The data presented here suggests an alteration in the CD8 T-cell compartment with impaired ability to control the EBV reactivation in COVID patients.

**Supplementary Information:**

The online version contains supplementary material available at 10.1007/s00430-026-00873-3.

## Introduction

The coronavirus disease 2019 (COVID-19) was initially considered as a self-limiting respiratory condition but later evidence show it may progress to a complex, multi-organ systemic illness with long-term consequences [1]. During the acute phase of COVID-19 the host immune system undergoes significant stress, lymphopenia, and a hyperinflammatory cytokine storm [2]. This state of transient immunodeficiency increases the risk of viral reactivation, particularly within the Herpesviridae family [3]. It is therefore important to understand the interplay between acute SARS-CoV-2-induced immune dysregulation and the reactivation of latent herpesviruses for efficient patient management [4].

Different reports have shown varying degrees of viral reactivation during acute COVID-19. Systematic reviews have identified the proportions of reactivation of members of the *Herpesviridae* to be Epstein–Barr virus (EBV): 45–58%, Human Herpesvirus (HHV)-1: 17.5–38%, Cytomegalovirus (CMV): 17.2–19%, and HHV-6: 5.3–18% [5, 6]. In a study of 173 consecutive cases, where 60 were SARS-CoV-2 positive, HHV-6B was identified in 13 out of 60 cases (21.7%) [7]. Another report found that HHV-6 co-occurrence was observed in 47.2% of COVID-19 patients, with a statistically significant association between HHV-6 detection and neurological changes [8]. Similarly, recent EBV reactivation was found associated with fatigue in PASC patients [9].

Acute viral reactivations may contribute to the complexities and emergence of Long COVID by establishing a state of persistent inflammation and immune exhaustion that lasts after clearance of the initial SARS-CoV-2 infection [4]. The lytic cycle of herpesviruses, particularly EBV and HHV-6, can induce ongoing mitochondrial dysfunction, microvascular damage, myocarditis, and the production of autoantibodies through molecular mimicry, creating a multi-layered pathology that manifests as the heterogeneous symptoms of PASC [3, 10–14]. EBV reactivation may increase ACE2 expression, which facilitates further SARS-CoV-2 viral entry and persistence, which in turn may favour EBV reactivation [15, 16].

Post-acute sequelae of COVID-19 (PASC), commonly termed Long COVID, has emerged as a significant health concern following the COVID-19 pandemic [17, 18]. The condition follows in at least 10% of the acute COVID-19 cases and is marked by a continuation of the initial acute COVID-19 symptoms, or emergence of new symptoms, more than four weeks after the initial SARS-CoV-2 infection [19, 20]. It is generally accepted that COVID-19 will become a seasonally recurring endemic disease with the potential of occasional epidemics [21]. This makes Long COVID a possible burden on health care systems for years to come.

A hallmark of PACS is fatigue, exertion intolerance, headache, myalgia, neurological and cognitive deficits as well as orthostatic disturbances, all which can severely impact the quality of life [22, 23]. Long term effects include an increased risk of adverse events, such as heart failure, deep vein thrombosis, end-stage renal disease, and acute kidney injury, with health deterioration up to 2 years after the initial infection [24, 25]. The underlying etiology for PASC is multifaceted with a persistent SARS-CoV-2 infection of the upper respiratory tract being one of many causes [26, 27].

While reactivation of EBV, CMV, and HHV-6 are frequently observed in severe COVID-19, they are not considered risk factors for severe COVID-19 or mortality [28–30]. EBV reactivation has been suggested to increase the risk for PASC [9, 31]. It has been proposed, that EBV may even be the causative agent for PASC, although a meta-analysis if 11 studies find the insufficient support for a definite conclusion [6, 32]. Although prior CMV infection is associated with a lower risk for the development of cognitive deficits, CMV and HHV-6 are less well described in PASC [9].

In this cross-sectional study, we investigate the mechanisms that underpin the relationship between acute COVID-19 and virus reactivation. We evaluated the composition of T cells in relation to reactivation of CMV, EBV, and HHV-6A/B in patients with at least COVID-19 of moderate severity at the time of hospitalization during the first wave of COVID-19. Our study concentrated on these viruses due to their classification within the Herpesviridae family and their well-documented ability to establish latent infections with potential for reactivation under specific circumstances. Previous infections with CMV, EBV, and HHV-6A/B were not an inclusion or exclusion criterion. Of the evaluated viruses, only EBV was overrepresented, irrespective of COVID-19 severity. Viral levels of EBV were negatively associated with CD4^+^ central memory T cell and negatively with CD8^+^ effector memory T cells in patients with Critical or Severe COVID-19, but not in Moderate COVID-19.

## Materials and methods

Patients admitted to University Hospitals in Essen and Bochum, Germany, with acute COVID-19 were recruited into the study in spring, 2020. A total of 61 patients were recruited, 33 were classified as Critical or Severe, and 28 were classified as moderate upon admission. The classification of COVID-19 manifestation was performed following Siddiqi and Mehra [33]. The study was approved by the Ethics Committee of the Ruhr University Bochum (20-6886) and University Hospital Essen (20-9214-BO). Written informed consent was obtained from all participants. Subjects were eligible for enrolment when they had a positive SARS-CoV2 PCR test and provided signed written informed consent. Prior infection with EBV, CMV, or HHV-6 was not a criterion. At the time of enrollment peripheral blood was collected in S-Monovette K3 EDTA blood collection tubes (Sarstedt). EDTA-treated whole blood was immediately subjected to immune cell phenotyping, and the remaining EDTA-treated whole blood was biobanked.

**Fig. 1:**
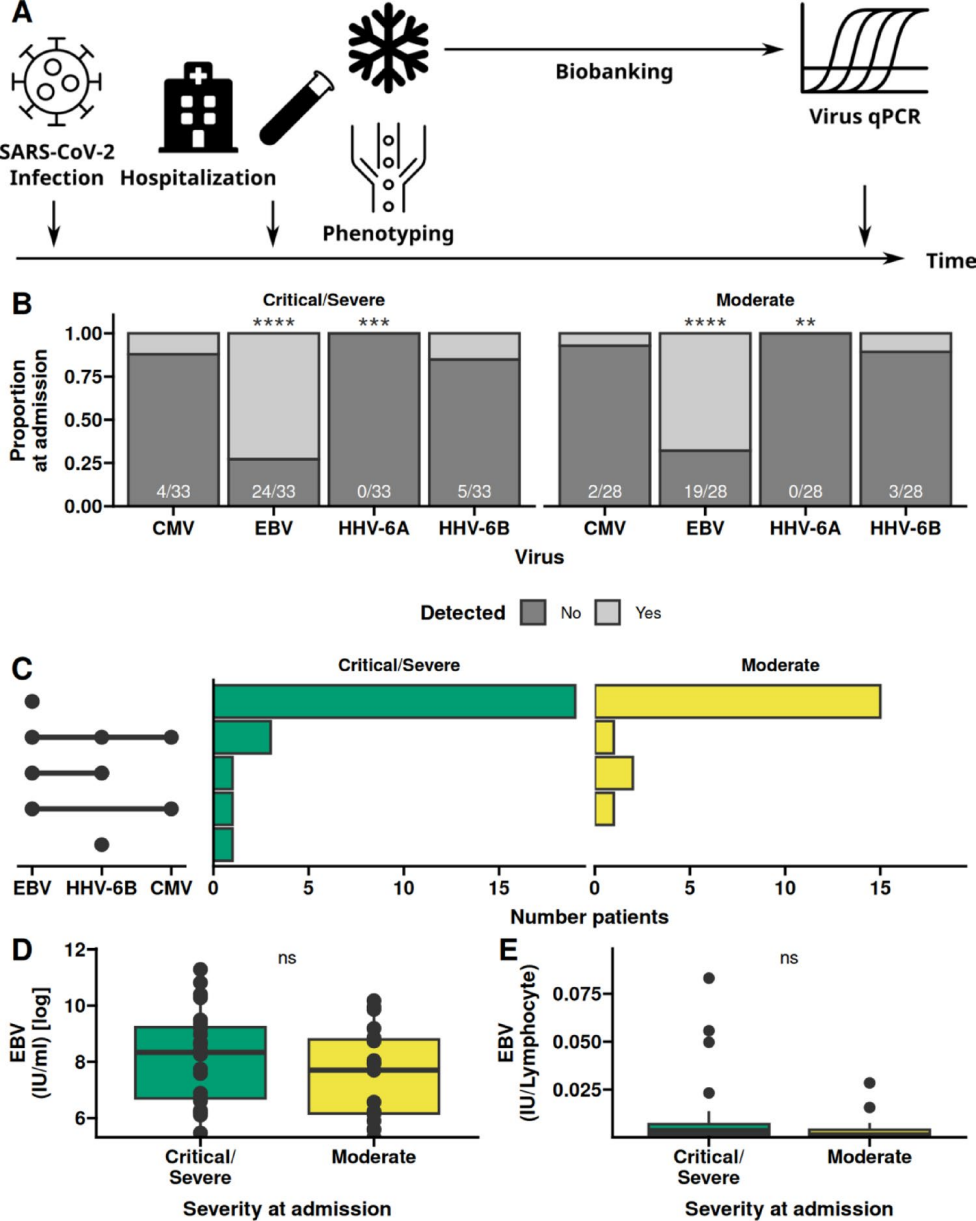
EBV is the most common co-infection irrespective of COVID-19 severity. Patients admitted to our hospital with a diagnosis of COVID-19 were classified following the criteria by Siddiqi and Mehra [33]. **A** Presence and absence of CMV, EBV, HHV-6A and 6B was detected by PCR over time in patients with at least moderate COVID-19 at admission. Over-representation of one of the two groups (reactivation/no reactivation) was assessed by Fisher’s exact test. **B** UpSet plot illustrating the co-occurrence of reactivated viruses. Left combination matrix gives the observed co-occurrence of the tested viruses, the bar graphs give the number of patients with the observed viral co-occurrence. **C** Viral load at admission. **D** Viral load per lymphocyte at admission. Box plots depict the median and the first and third quartiles. The whiskers correspond to 1.5 times the interquartile range. Differences in C and D were assessed by the ANOVA with correction for age and sex

### Quantification of EBV, CMV, HHV-6A, and HHV-6B

DNA was isolated from whole blood using the QIAamp DNA Blood Mini Kit (Qiagen). EBV was quantified using the RealStar EBV Kit 1.0, CMV was quantified using the RealStar CMV kit 1.0, and HHV-6A and HHV-6B were quantified using the RealStar HHV-6 kit 1.0. All RealStar kits were purchased from Altona Diagnostics and were used per manufacturer’s instructions. The RealStar EBV Kit 1.0, the RealStar CMV kit 1.0 and the RealStar HHV-6 kit 1.0 (all purchased from Altona Diagnostics) were used according to manufacturer’s instruction to quantify the viral load of EBV, CMV and HHV-6A/B, respectively. The RealStar HHV-6 kit 1.0 allows discrimination and quantification of HHV-6A and HHV-6B specific DNA. The HHV6-B detection system of the RealStar HHV-6 kit 1.0 may also detect some HHV6-A strains through cross reactivity. In such cases a regular strong signal for HHV-6A is seen together with a weak HHV-6B signal, due to inefficient amplification by mismatching HHV-6B primers. In all cases, a HHV6-B signal with a ct > 38 was classified as negative. To stay within an analytical sensitivity rate of 100% detected virus specific DNA for each test system, we defined a detection limit of 250 copies/ml. Patients with viral load over the detection limit for were defined as positive for the corresponding virus.

### Antibodies

All used antibodies, including vendor and relevant Research Resource Identifiers (RRID) are listed in Table 1. The phenotyping panel was composed of CD45-A488; CD56-PerCP-Cy5.5; CD14-PE-Vio 770; CD4-A700; CD16-APC-Vio 770; CD8-V500; CD19-BV605; HLA-DR-BV650; CD3-BV785. The gating strategy is given in Figure S1. The T cell subsets staining panel was composed of CCR7-PE; CD127-PC7; CD25-FITC; CD3-APC-750; CD45RA-Pacific Blue; CD4-ECD; CD8-APC; TCRα/β-PerCP-Cy5.5; TCRγ/δ-BV510. The gating strategy is given in Figure S2. The staining panel for T cell activation state ex vivo was composed of CD11a-FITC; CD28- PerCP-Cy5.5; CD57-Pacific Blue; CD3-APC-750; HLA-DR-PE; CD4-ECD; CD8-APC. The gating strategy is given in Figure S3.

**Table 1:** Antibody details

Antigen	Fluorophore	Clone	Manufacturer	Cat#	RRID
CCR7	PE	G043H7	Beckman Coulter	B30632	–
CD11a	FITC	25.3	Beckman Coulter	IM0860U	–
CD127	PC7	R34.34	Beckman Coulter	A64618	AB_2833031
CD14	PE-Vio 770	TÜK4	Miltenyi Biotec	130-113-149	AB_2725977
CD16	APC-Vio 770	REA423	Miltenyi Biotec	130-113-390	AB_2733101
CD19	BV605	HIB19	BioLegend	302,244	AB_2562015
CD25	FITC	B1.49.9	Beckman Coulter	IM0478U	AB_130985
CD28	PerCP-Cy5.5	CD28.2	Beckman Coulter	B24027	–
CD3	BV785	OKT3	BioLegend	317,330	AB_2563507
CD3	APC-750	UCHT1	Beckman Coulter	A94680	–
CD4	AF700	OKT4	BioLegend	317,426	AB_571943
CD4	ECD	SCF4I12T4D11	Beckman Coulter	6,604,727	AB_2833032
CD45	AF488	2D1	BioLegend	368,536	AB_2721364
CD45RA	Pacific Blue	2H4	Beckman Coulter	A82946	AB_2904200
CD56	PerCP-Cy5.5	NCAM	BioLegend	304,626	AB_10641700
CD57	Pacific Blue	NC1	Beckman Coulter	A74779	–
CD8	V500	RPA-T8	Becton Dickinson	560,774	AB_1937333
CD8	APC	B9.11	Beckman Coulter	IM2469	AB_130782
HLA-DR	BV650	L243	BioLegend	307,650	AB_2563828
HLA-DR	PE	Immu-357	Beckman Coulter	IM1639	AB_131284
TCRα/β	PerCP-Cy5.5	IP26	BioLegend	306,724	AB_2563002
TCRγ/δ	BV510	B1	BioLegend	331,220	AB_2564275

### Flow cytometry

Samples were prepared for flow cytometry as previously described [34]. Briefly: EDTA-treated whole blood was stained with optimal concentrations of each antibody for 10 min at room temperature in the dark. Erythrocytes were lysed using VersaLyse (Beckman Coulter) with 2.5% IOTest 3 fixative solution (Beckman Coulter) for 30 min at room temperature in the dark. Samples for general phenotyping were immediately acquired after staining, while samples for T and B cell subsets were washed twice with PBS/BSA after staining and before acquisition (Table 2).

**Table 2:** Tool details

Tool	Version	Vendor	RRID
FlowJo	10.6.2	BD	SCR_008520
R	4.3.3	R Foundation for Statistical Computing	SCR_001905
CytoFlex	–	Beckman Coulter	SCR_019627

All samples were acquired on a CytoFLEX flow cytometer (Beckman Coulter). Quality control was performed daily using the recommended CytoFLEX daily QC fluorospheres (Beckman Coulter). No modification to the compensation matrices was required throughout the study. See Table S2 for relevant RRIDs.

### Statistics

Flow cytometry data were analyzed using FlowJo (version 10.6.2, BD Biosciences). Differences between groups were assessed using the Wilcoxon rank sum test and ANOVA with correction for age and sex for comparisons between COVID-19 severity, using R, version 4.3.3 (R Foundation for Statistical Computing). See Table S2 for relevant RRIDs. *p* values were not corrected for multiple testing, as this study was of an exploratory nature [35, 36]. Box plots depict the median, first quantile, and third quartile of a variable; the whiskers correspond to 1.5 times the interquartile range.

## Results

### Incidence rates of EBV, CMV, and HHV-6 in COVID-19

During the initial wave of COVID-19 in early 2020, several patients with a disease severity of moderate or worse were recruited into the study (Table 3). A total of 61 patients were recruited; 28 patients were classified upon admission as having a Moderate COVID-19 and 33 were classified as having a Critical or Severe disease. This cohort gives the opportunity to gain insights into the interplay between COVID-19 and other infections. Blood was obtained from the enrolled patients upon admission and subjected to qPCR to detect Cytomegalovirus (CMV), Epstein–Barr Virus (EBV), Human Herpesvirus (HHV)-6A and HHV-6B (Fig. 1A). We found an over-representation of EBV-reactivation irrespective of COVID-19 severity (24 of 33 (72.7%); *p*: < 0.001, 19 of 28 (67.9%); *p*: < 0.001; Fisher’s exact test). HHV-6A was not detected among any patients, and the proportion of CMV (4 of 33 (12.1%) and 2 of 28 (7.1%)) and HHV-6B (5 of 33 (15.2%) and 3 of 28 (10.7%)) were insignificant (Fig. 1B). In most of the patients with virus reactivation, only EBV could be detected, but also co-occurrence could be observed (Fig. 1C). There was a tendency to higher viral load of EBV in COVID-19 patients with a critical or severe COVID-19 disease (Fig. 1D). Lymphopenia is a common feature of COVID-19 [37], but normalizing the viral load to the lymphocyte count, did not alter the observed differences in viral loads between the patient groups (Fig. 1E). Similar observations were made for CMV and HHV-6B (Figure S4). Collectively, EBV reactivation was found not only in severe COVID-19 cases but also in patients with moderate disease at the time of hospital admission. In contrast, the prevalence of CMV and HHV-6A and -6B was low.

**Table 3:** Characteristics of patient stratification

COVID-19				
	Critical/Severe	Moderate	*p*	Test
n	33	28		
Sex = Male (%)	24 (72.7)	11 (39.3)	0.011	Fischer test
Age (median [IQR])	65.60 [56.40, 79.30]	52.25 [38.83, 62.80]	0.005	Kruskal–Wallis

EBV				
	EBV^−^	EBV^+^	*p*	
n	18	43		
Sex = Male (%)	11 (61.1)	24 (55.8)	0.781	Fischer test
Age (median [IQR])	57.95 [43.02, 61.22]	60.50 [48.50, 76.10]	0.258	Kruskal–Wallis

CMV				
	CMV^−^	CMV^+^	*p*	
n	55	6		
Sex = Male (%)	30 (54.5)	5 (83.3)	0.227	Fischer test
Age (median [IQR])	58.90 [47.85, 74.30]	57.15 [47.48, 67.65]	0.981	Kruskal–Wallis

HHV-6B				
	HHV-6B^−^	HHV-6B^+^	*p*	
n	53	8		
Sex = Male (%)	27 (50.9)	8 (100.0)	0.016	Fischer test
Age (median [IQR])	60.50 [51.00, 76.80]	50.90 [43.88, 58.25]	0.109	Kruskal–Wallis

Stratifying the cohort based on EBV, CMV or HHV-6B revealed no significant differences in age or biological sex (Table 3). Only exception was HHV-6B where all 8 HHV-6B positive COVID-19 patients were male.

### T-cell phenotype in EBV reactivation

**Fig. 2:**
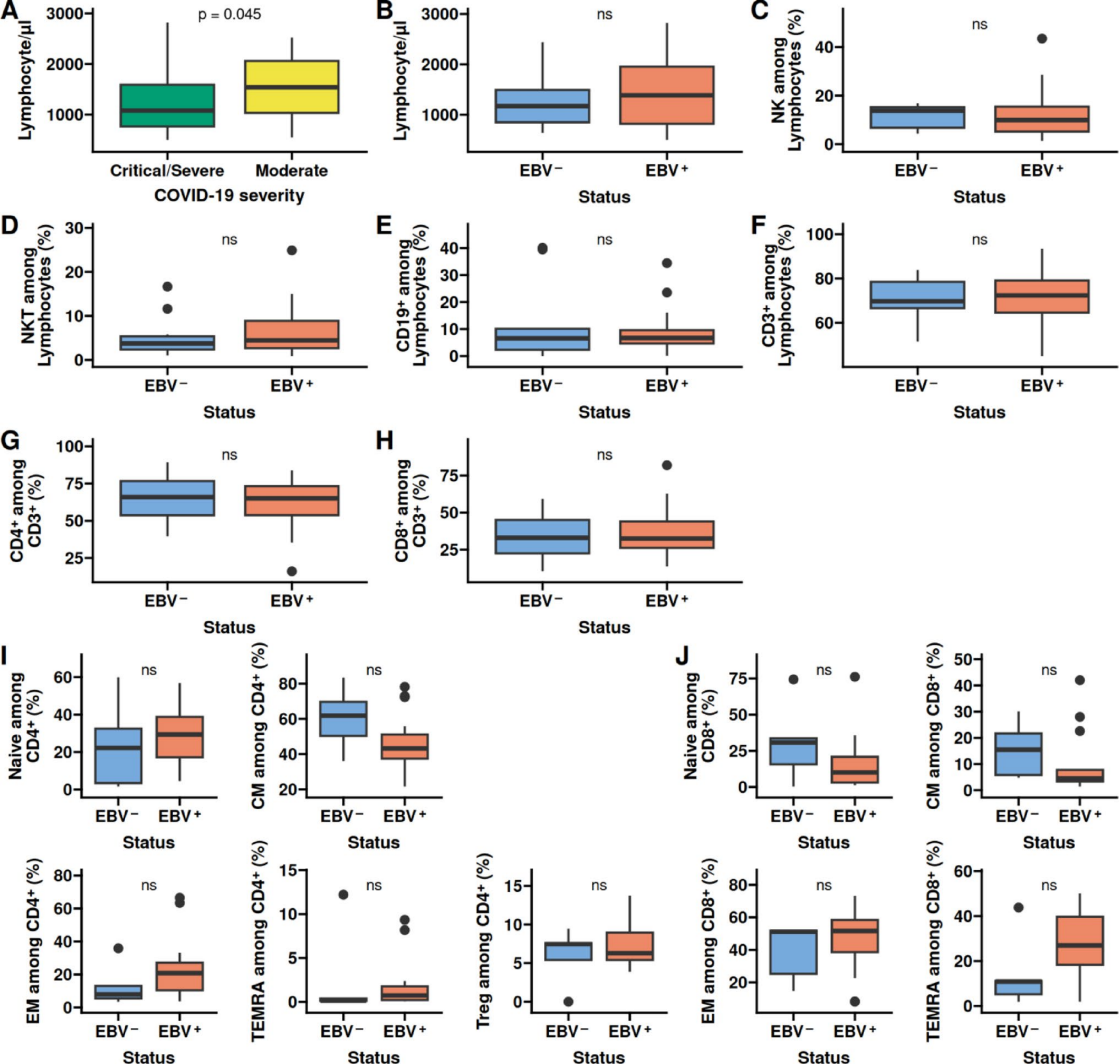
Major lymphocyte populations are similar. Whole blood collected from the patients was evaluated for all major immune cell populations as well as memory subsets (Naive (CD45RA^+^CCR7^+^), Central memory (CD45RA^−^CCR7^+^), Effector memory (CD45RA^−^CCR7^−^), and TEMRA (CD45RA^+^CCR7^−^)) in CD4^+^ and CD8^+^ T cells. **A** Lymphocyte count between Critical/Severe and moderate COVID-19 severity. **B** Lymphocyte count in patients with and without EBV reactivation. **C** Frequency of NK cells in patients with and without EBV reactivation. **D** Frequency of NKT cells in patients with and without EBV reactivation. **E** Frequency of CD19 positive B cells in patients with and without EBV reactivation. **F** Frequency of CD3^+^ T cells in patients with and without EBV reactivation. **G** Frequency of CD4^+^ T cells in patients with and without EBV reactivation. **H** Frequency of CD8^+^ T cells in patients with and without EBV reactivation. **I** Naive (CD45RA^+^CCR7^+^), Central memory (CD45RA^−^CCR7^+^), Effector memory (CD45RA^−^CCR7^−^), and TEMRA (CD45RA^+^CCR7^−^) in CD4^+^ T cells. **J** Naive (CD45RA^+^CCR7^+^), Central memory (CD45RA^−^CCR7^+^), Effector memory (CD45RA^−^CCR7^−^), and TEMRA (CD45RA^+^CCR7^−^) in CD4^+^ T cells. CM: Central memory. EM: Effector memory. TEMRA: T Effector memory expressing CD45RA. Box plots depict the median and the first and third quartiles. The whiskers correspond to 1.5 times the interquartile range. Difference is A was assessed by the ANOVA with correction for age and sex. All other comparisons were assessed by the Wilcox test

We found weakly significant decreased lymphocyte counts in patients with critical or severe COVID-19 disease after adjusting for age and sex (*p* = 0.045; Fig. 2A). Age was a significant predictor (*p* = 0.012), while sex was not (*p* = 0.24). The difference in lymphocyte counts was not present when comparing patients with and without EBV reactivation, irrespective of COVID-19 disease course (Fig. 2B). Further, there was no difference in any of the examined major immune cells populations NK, NKT, CD19, CD3, CD4, or CD8 (Fig. 2C–H). Differences in the overall frequency of the subtypes Naive (CD45RA^+^CCR7^+^), Central memory (CD45RA^−^CCR7^+^), Effector memory (CD45RA^−^CCR7^−^), and TEMRA (CD45RA^+^CCR7^−^) in CD4^+^ (Fig. 2I) and CD8^+^ (Fig. 2J) also were not associated with EBV reactivation. For HHV-6B^+^ we found a decrease in NK populations and an increase in Treg (Figure S5A and I). The central memory population in CMV^+^ patients was significantly reduced on both CD4^+^ and CD8^+^ T-cells (Figure S5I and J). Taken together, these data indicate that all major lymphocyte populations are similar in COVID-19 patients irrespective of EBV reactivation.

### Mechanisms linking acute COVID-19 and EBV reactivation

**Fig. 3:**
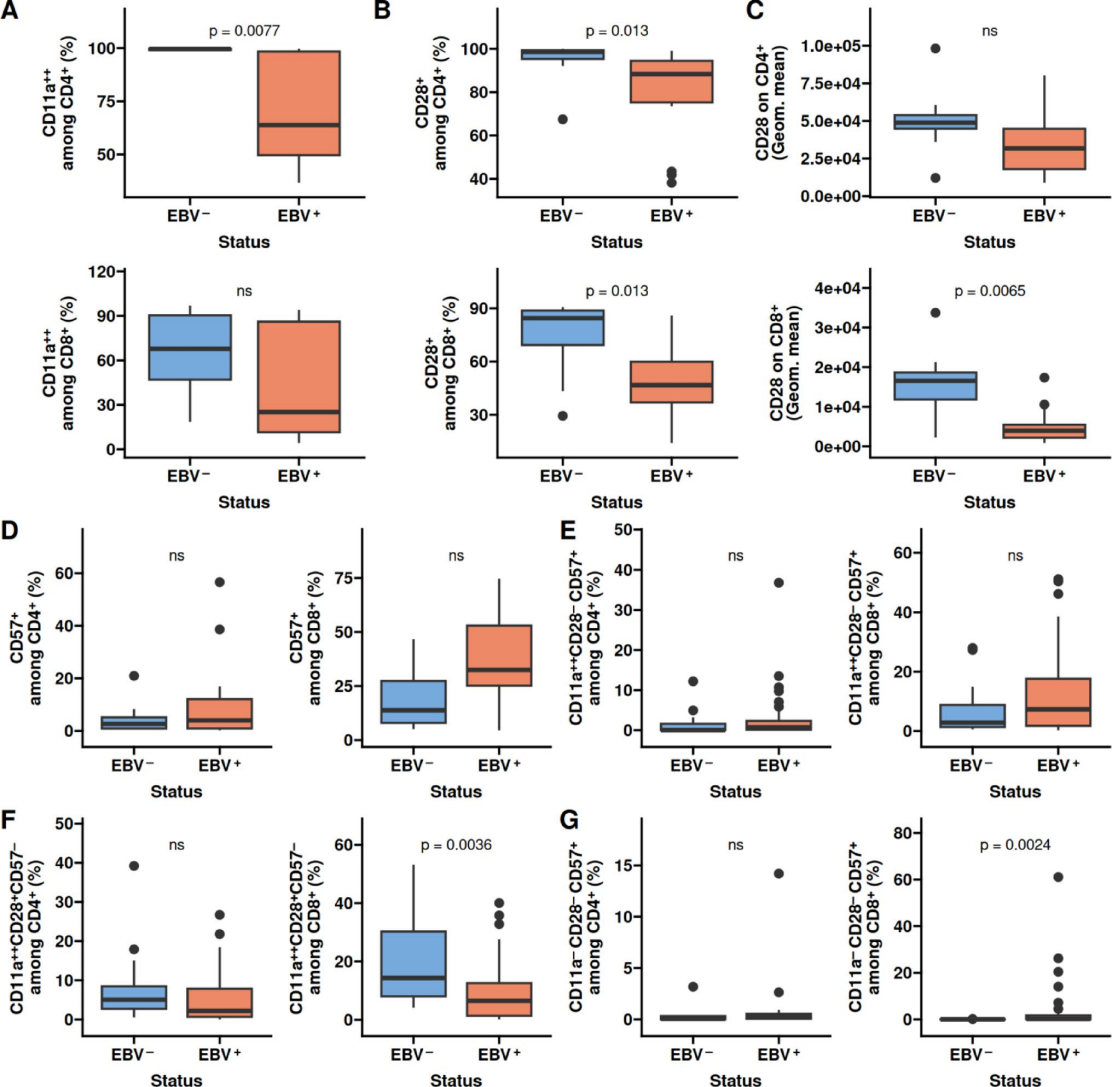
Decreased T cell activation markers and increased replication senescence in CD8 T cells. The collected whole blood was evaluated for surface expressed activation markers. **A** Frequency of CD11a among CD4 T cells (top) and CD8 T cells (bottom). **B** Frequency of CD28 among CD4 T cells (top) and CD8 T cells (bottom). **C** Frequency of CD57 among CD4 T cells (top) and CD8 T cells (bottom). **D** Geometric meaning of CD28 on CD4 T cells (left) and CD8 T cells (right). **E** Frequency of CD11a^++^CD28^+^CD57^−^ CD4 T cells (left) and CD8 T cells (right). **F** CD11a^−^CD28^−^CD57^+^ among CD4 T cells (right) and CD8 T cells (left). Box plots depict the median and the first and third quartiles. The whiskers correspond to 1.5 times the interquartile range. Difference was assessed by the Wilcox test

The integrin CD11a is important for transmigration of the T-cells across the endothelial barrier and activated T-cells increase expression of CD11a. Our previous studies have shown a decrease in frequency of CD11a^++^ T cells in patients with severe COVID-19 [34]. We wondered if a similar effect could be observed for patients with EBV reactivation and found that EBV is associated with a decreased frequency of CD11a^++^  among CD4^+^ T cells (*p* = 0.0077), but not CD8^+^ T cells (Fig. 3A). Given the lack of activated T-cells, we were surprised to also observe a decrease of CD28 among both CD4^+^ and CD8^+^ T cells (*p* = 0.013 and 0.013, respectively; Fig. 3B), which is also associated with T-cell activation. Not only did we observe a decrease in frequency of CD28^+^ T cells, but also the degree of expression on each CD8^+^ T cell expressing CD28 was decreased (*p* = 0.0065; Fig. 3C).

CD57 is a known surface molecule associated with replicative senescence and a stable TEMRA phenotype [38–40]. We observed a tendency to increased expression in patients with EBV reactivation, although it did not reach statistical significance (Fig. 3D). When combining this through Boolean gating, there was a tendency for increased frequencies CD11a^++^CD28^−^CD57^+^ among CD4^+^ and CD8^+^ T cells (Fig. 3E). We also evaluated the co-expression of CD11a and CD28 or CD57 in the absence of CD11a and CD28. We found a tendency for a decreased frequency of CD11a^++^CD28^+^CD57^−^ among CD4^+^ T cells, which was significant for CD8^+^ T-cells (*p* = 0.0036; Fig. 3F). CD11a^−^CD28^−^CD57^+^ was slightly increased among CD4^+^ but significantly increased among CD8^+^ T-cells (*p* = 0.0024; Fig. 3G).

No differences were observed for either CMV or HHV-6B (Figure S6). Collectively, patients with EBV reactivation exhibited an altered activation phenotype and higher replicative senescence in circulating CD8 T cells. Specifically, there was diminished expression of the T-cell co-stimulatory CD28 and co-expression of CD28 and the integrin CD11a in CD8 T cells, along with increased frequency of CD8 T cells expressing the proliferative exhaustion marker CD57.

## Discussion

In the present cross-sectional study, we investigated the relationship between acute COVID-19 and reactivation of common latent viruses such as Epstein–Barr Virus (EBV), Cytomegalovirus (CMV), and Human Herpesvirus (HHV)-6A and -6B. These viruses are all members of the Herpesviridae family and are known to establish latent infections with reactivation under certain conditions. The study was incited by previous observations of association between EBV reactivation and PASC symptoms [6, 27, 41, 42]. We therefore wondered if other viruses of the same family may behave in a similar fashion. We found that at the time of hospital admission EBV was reactivated in patients with moderate to severe COVID-19, irrespective of severity. In contrast, the other examined persisting viruses CMV and HHV-6A and -6B had only a low prevalence in the patients. The reactivation of EBV was accompanied by lower expression of CD28 and CD11a, but increased expression of CD57 on cytotoxic CD8^+^ T cells.

With an incidence of 67.9–72.7% for EBV reactivation among the COVID-19 patients, the cohort of this study had higher incidences than previously observed [5, 6]. The incidence rate of HHV-6B reactivations of 10.7–15.2% was also lower than previous observations, where the rate was found to be 21.7–47.2% [7, 8], and only the observed incidence of CMV was similar to previous reports [5, 6]. The reasons for the discrepancies are not clear but may be due to geographical and demographic factors.

No significant variations were observed in the cell populations examined between SARS-CoV-2 patients with EBV reactivation and those without. We extended the analysis to include combinations of the surface molecules CD11a, CD28, and CD57 in CD4^+^ and CD8^+^ T-cells. CD11a is a surface molecule found on all leukocytes and facilitates their extravasation though binding to adhesion molecules like ICAM-1, and the frequency of CD11a^++^ T-cells is increased in acute EBV infection [43]. Signaling through the co-stimulation molecule CD28 protects activated T-cells by upregulation of anti-apoptotic proteins [44]. Consequently, diminished levels of CD28 were reported to correlate with a state of acute T-cell depletion [45]. Conversely, the increased CD57 expression on T cells is a marker of terminal differentiation of chronically stimulated T cells. CD57^+^ T-cells have reduced proliferation potential, resistance to apoptosis, and an enhanced cytotoxic potency [38, 46].

We found a tendency for increased frequencies of CD11a^++^CD28^−^CD57^+^ in both T-cell populations, indicative of an activation of T cells in patients with EBV reactivation. This is further supported by a decreased prevalence of CD28^+^ CD8^+^ T cells. There was a reduced occurrence of CD28^+^CD11a^+^CD57^−^CD8^+^ T cells, alongside an elevation in the frequency of CD28^−^CD11a^−^CD57^+^ CD8^+^  T cells. CD28^−^CD57^+^ CD8^+^ T-cells have been shown to upregulate expression of PD-1 and a to display a reduced killing of EBV infected cells [47]. This indicates a decreased anti-EBV potential in patients with EBV-reactivation. CD28^−^CD57^+^ in COVID-19 patients could be driven by CMV, as previously observed [48]. However, given the low degree of CMV activation in our cohort, this is likely not the explanation for our observations. Collectively, the observed reduction in recently activated CD28 and CD11a expressing CD8 T-cells and increased number of CD8^+^CD57^+^ T cells with or without CD11a^++^ indicate increased exhaustion with an atypical phenotype in SARS-CoV-2 patients with EBV reactivation. These findings are consistent with recent evidence suggesting that the immune control of EBV is heavily dependent on early-differentiated T cells [49]. While the data presented here highlight a shift toward terminal differentiation and exhaustion (CD28^−^CD57^+^), it remains unclear whether these phenotypic changes specifically affect EBV-specific T-cell populations or represent a broader bystander effect of the acute COVID-19 inflammatory environment. CD27 is another marker of T-cell differentiation alongside CD28. The loss of CD27 characterizes the transition from early to late differentiation, and it remains to be determined how this molecule associates with EBV reactivation and Long COVID [50].

Reactivation of viruses like EBV have been observed in PASC patients and in patients with ME/CFS [51, 52]. Our observed incidence of virus reactivation is independent of COVID-19 severity which matches the notion that PASC is independent of COVID-19 severity [19]. The observed increased expression of CD57, which associated with expression of PD-1, makes a connection between EBV reactivation and PASC, where an increase of exhaustion markers PD-1 and TIM-3 have been reported [53, 54].

Given the exhaustion of CD8^+^ T cells in COVID-19 patients with EBV reactivation, it is curious that none of the other examined viruses were detected in a significant proportion of the patients. CMV infection results in a decreased diversity of the EBV-specific T-cell repertoire [55]. Also, functionality may be altered, as EBV-specific T cells were found to have disproportionate expression of genes involved in synapse formation compared to CMV specific T-cells [56]. It has further been reported that EBV may be directly or indirectly activated by a variety of pathogens, including CMV [57]. Collectively, this makes it feasible that CMV indeed is reactivated in patients with acute COVID-19, but that this is better controlled, so only few patients with measurable levels of CMV could be identified.

The study focuses on the direct mechanism of EBV reactivation in patients with acute COVID-19. This cross-sectional design makes it impossible to determine whether reactivation of latent viruses directly contributes to post-acute sequelae of COVID-19. Furthermore, the majority of the patients in our cohort were lost to follow-up, which prevented us from identifying which individuals went on to develop PASC or assessing if specific Long COVID symptoms, previously associated with altered EBV infection, were present in this group [9]. The observed reactivation of latent viruses could alternatively be the result of other factors unrelated to COVID-19, such as pre-existing health conditions. However, our observation that virus reactivation occurred independently of COVID-19 severity aligns with the clinical notion that the development of PASC is often independent of the initial acute disease severity [19]. The same limitation holds for the observed altered phenotype; we cannot establish if this is the reason for EBV reactivation, or a result thereof. The study size, with its 61 patients, is also relatively small and patients came from a relatively small geographical region, which may limit the generalization of the findings.

Our analysis of viral dynamics has certain limitations. While we detected herpesvirus by presence of DNA in peripheral blood, we could not assert the serological state against the same viruses. The data presented here may therefore underestimate the degree of reactivation, especially for low levels of reactivation. Additionally, the SARS-CoV-2 testing was recorded qualitatively as positive/negative, which excludes a direct correlation between SARS-CoV-2 viral loads and EBV reactivation levels. Such comparison may elucidate the underlying mechanism: transient general or pathogen specific immune impairment.

The data presented in this study suggests that in patients with acute COVID-19, EBV reactivation is associated with an altered CD8 T-cell activation phenotype, coupled with exhaustion.

## Supplementary Information

Below is the link to the electronic supplementary material.

Supplementary Material 1

## Data Availability

The data that support the findings of this study are available from the corresponding author, NB, upon reasonable request.
