# Supplementary material for: Acute COVID-19 is associated with altered CD8 T-cells indicative of impaired ability to control Epstein–Barr virus reactivation
Source: Med Microbiol Immunol. 2026 Apr 20;215(1):13. doi: 10.1007/s00430-026-00873-3 (PMC13092548; doi:10.1007/s00430-026-00873-3)
Supplement: Supplementary file 1 — Supplementary Material 1 [file 430_2026_873_MOESM1_ESM.pdf]

Acute COVID-19 is associated with altered CD8 T-cells indicative of impaired ability to control Epstein-Barr Virus Reactivation – Supplement

Ulrik Stervbo[1,2], Moritz Anft[1], Krystallenia Paniskaki[3], Arturo Blazquez-Navarro[2], Adrian Doevelaar[1], Sarah Skrzypczyk[1], Eva Kohut[1], Julia Kurek[1], Patrizia Wehler[2], Sviatlana Kaliszczyk[1], Kamil Rosiewicz[1], Felix S. Seibert[1], Bodo Hölzer[1], Constantin J. Thieme[4], Toralf Roch[2], Margarethe Justine Konik[3], Marc Moritz Berger[5], Thorsten Brenner[5], Uwe Kölsch[6], Michael Adamzik[7], Michael Schmueck-Henneresse[2], Carmen Scheibenbogen[4,6], Sebastian Dolff[3], Ulf Dittmer[8], Oliver Witzke[3], Timm H. Westhoff[1], Nina Babel[1,2]

1. Center for Translational Medicine and Immune Diagnostics Laboratory, Medical Department I, Marien Hospital Herne, University Hospital of the Ruhr University Bochum, Hölkeskampring 40, 44625 Herne, Germany
2. Berlin Institute of Health (BIH) at Charité – Universitätsmedizin Berlin, BIH Center for Regenerative Therapies (BCRT), Experimental Immunotherapy, Augustenburger Platz 1, 13353 Berlin, Germany
3. Department of Infectious Diseases, West German Centre of Infectious Diseases, University Hospital Essen, University Duisburg-Essen, Hufelandstraße 55, 45147 Essen, Germany
4. Charité – Universitätsmedizin Berlin, Corporate Member of Freie Universität Berlin, Humboldt-Universität zu Berlin, Berlin Center for Advanced Therapies, Augustenburger Platz 1, 13353 Berlin, Germany
5. Department of Anesthesiology and Intensive Care Medicine, University Hospital Essen, University Duisburg-Essen, Hufelandstraße 55, 45147 Essen, Germany
6. Department of Immunology, Labor Berlin GmbH, Sylter Straße 2, 13353 Berlin, Germany
7. Clinic for Anesthesiology, Intensive Care Medicine and Pain Therapy, University Hospital Knappschaftskrankenhaus Bochum, In der Schornau 23-25, 44892 Bochum, Germany
8. Institute for Virology, University Hospital Essen, University of Duisburg-Essen, Hufelandstraße 55, 45147 Essen, Germany

Correspondence

Nina Babel, nina.babel@charite.de

Table of Contents

Tables....................2

Figures....................3

References....................5

Figures

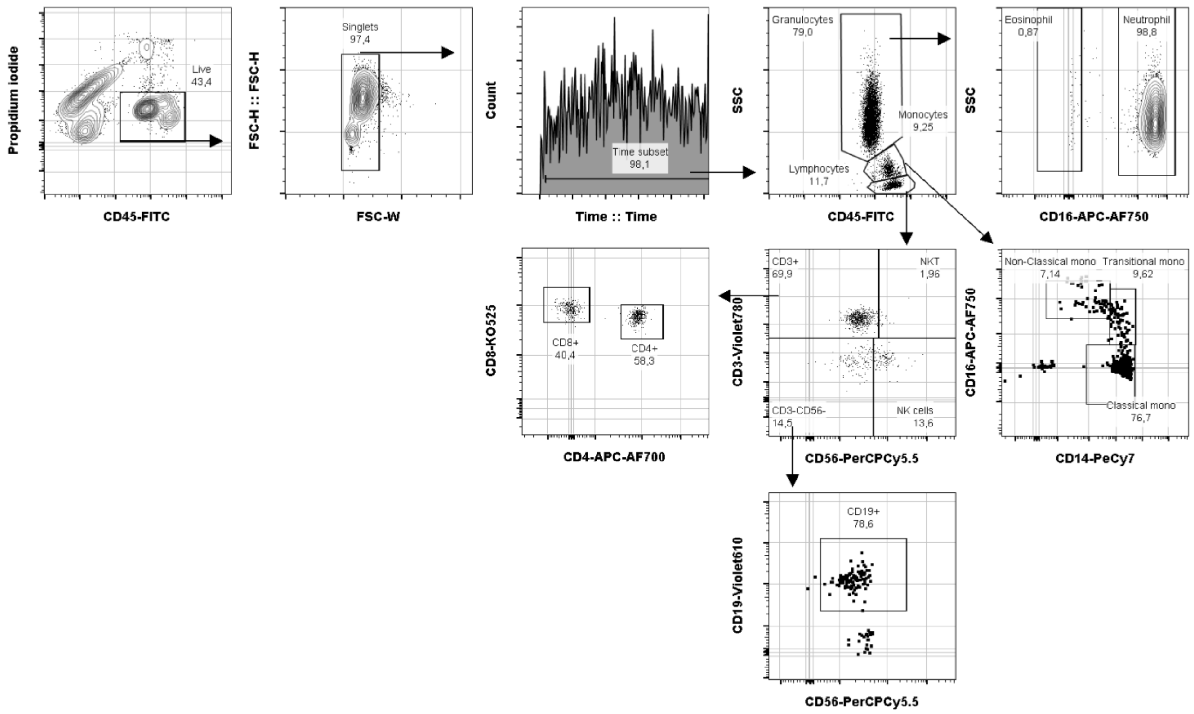

Supplementary Figure 1. Gating strategy for general phenotyping.

Whole blood was diluted 1:1 in staining solution prior to erythrocytes lysis. Live cells were identified as negative for propidium iodide and doublets were excluded. Granulocytes, monocytes and lymphocytes were distinguished by CD45 and side scatter profile. Granulocytes were separated by CD16 in Eosinophiles (CD16-) and Neutrophiles (CD16+). Monocytes were divided by the expression of CD14 and CD16 into classical- (CD14++CD16-), intermediate- (CD14++CD16+) and non-classical Monocytes (CD14+CD16+). Lymphocytes were separated by CD3 and CD56 in NK (CD3-CD56+), NKT (CD3+CD56+) and T cells (CD3+CD56-) and T cells were further distinguished into CD4+ helper T cells and CD8 + cytotoxic T cells. B cells were identified as CD3/CD56 double negative and CD19 positive cells.

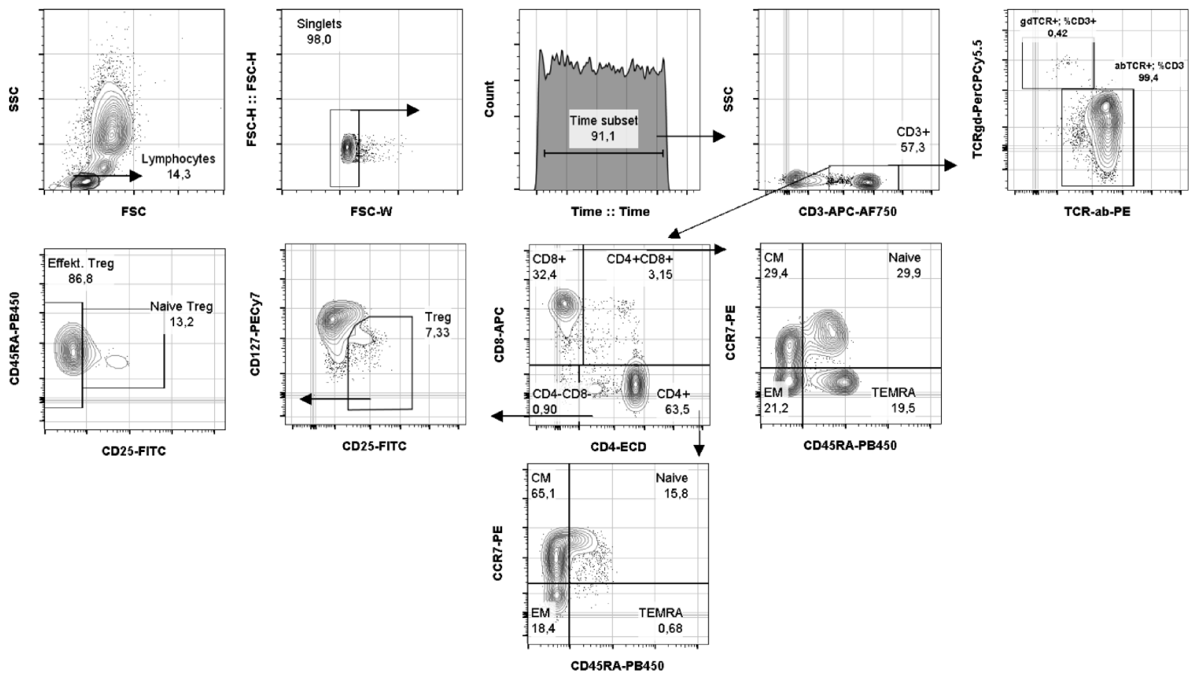

Supplementary Figure 2. Gating strategy for T cell subsets.

Whole blood was diluted 1:1 in staining solution prior to erythrocytes lysis. Living lymphocytes were identified by their forward and sideward scatter profile and doublets were excluded. T cells were identified by the expression of CD3 and separated into αβ-T cells and γδ-T cells by staining with TCR antibodies against TCRαβ and TCRγδ. Additionally, CD3+ T cells were divided into T-helper cells (CD4+CD8-) and cytotoxic T cells (CD4-CD8+). Both T cell subsets were further divided by the expression of CD45RA and CCR7 into naïve (CD45RA+ CCR7+), central memory (CM, CD45RA-CCR7+), effector memory (EM, CD45RA-CCR7-) and T effector RA (TEMRA, CD45RA+CCR7-) T cells. Additionally, regulatory T cells (Tregs) were identified as CD4+CD25+CD127+ and further separated by their expression of CD45RA into Naïve (CD45RA +) and effector-Tregs (CD45RA-).

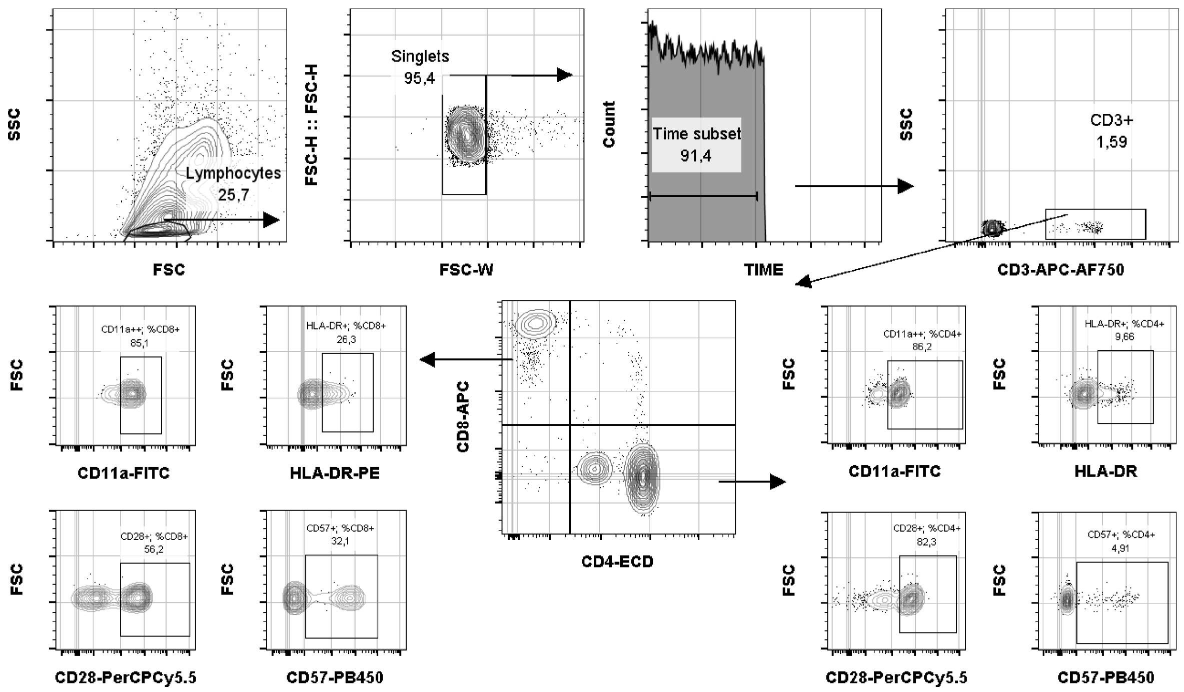

Supplementary Figure 3. Gating strategy for the T cell activation state ex vivo.

Whole blood was diluted 1:1 in staining solution prior to erythrocytes lysis. Living lymphocytes were identified by their forward and sideward scatter profile and doublets were excluded. T cells were identified as CD3 and separated by expression of CD4 and/or CD8 into double negative (CD4-CD8-), double positive (CD4+CD8+), T-helper cells (CD4+CD8-) and cytotoxic T cells (CD4-CD8+). The expression of activation marker CD11a, HLA-DR, CD28 and CD57 was assessed on CD4+ and CD8+ single positive T cells.

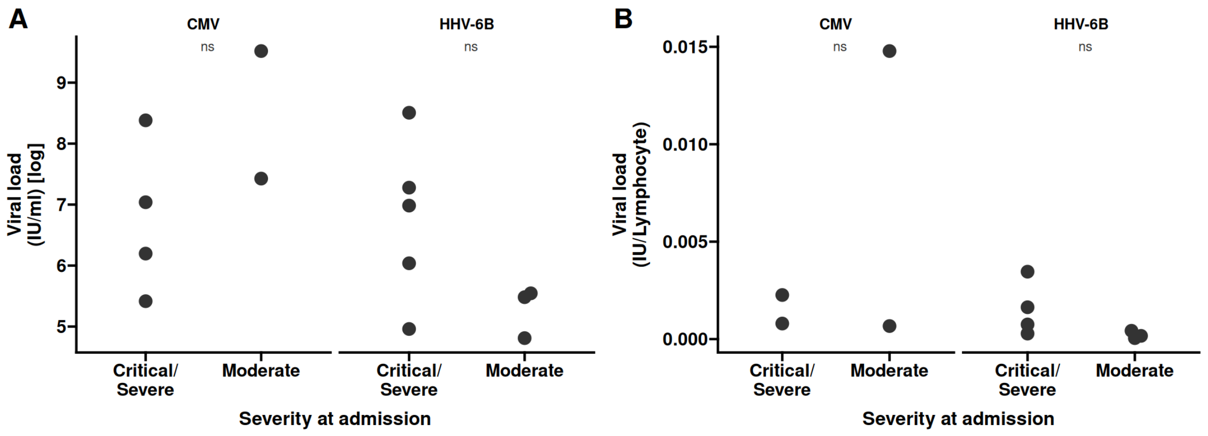

Supplementary Figure 4. Viral load at admission.

Patients admitted to our hospital with COVID-19 diagnose were classified following the criteria by Siddiqi and Mehra (Siddiqi and Mehra 2020). A) Viral load at admission. B) Viral load per lymphocyte at admission. Difference was assessed by Wilcox test.

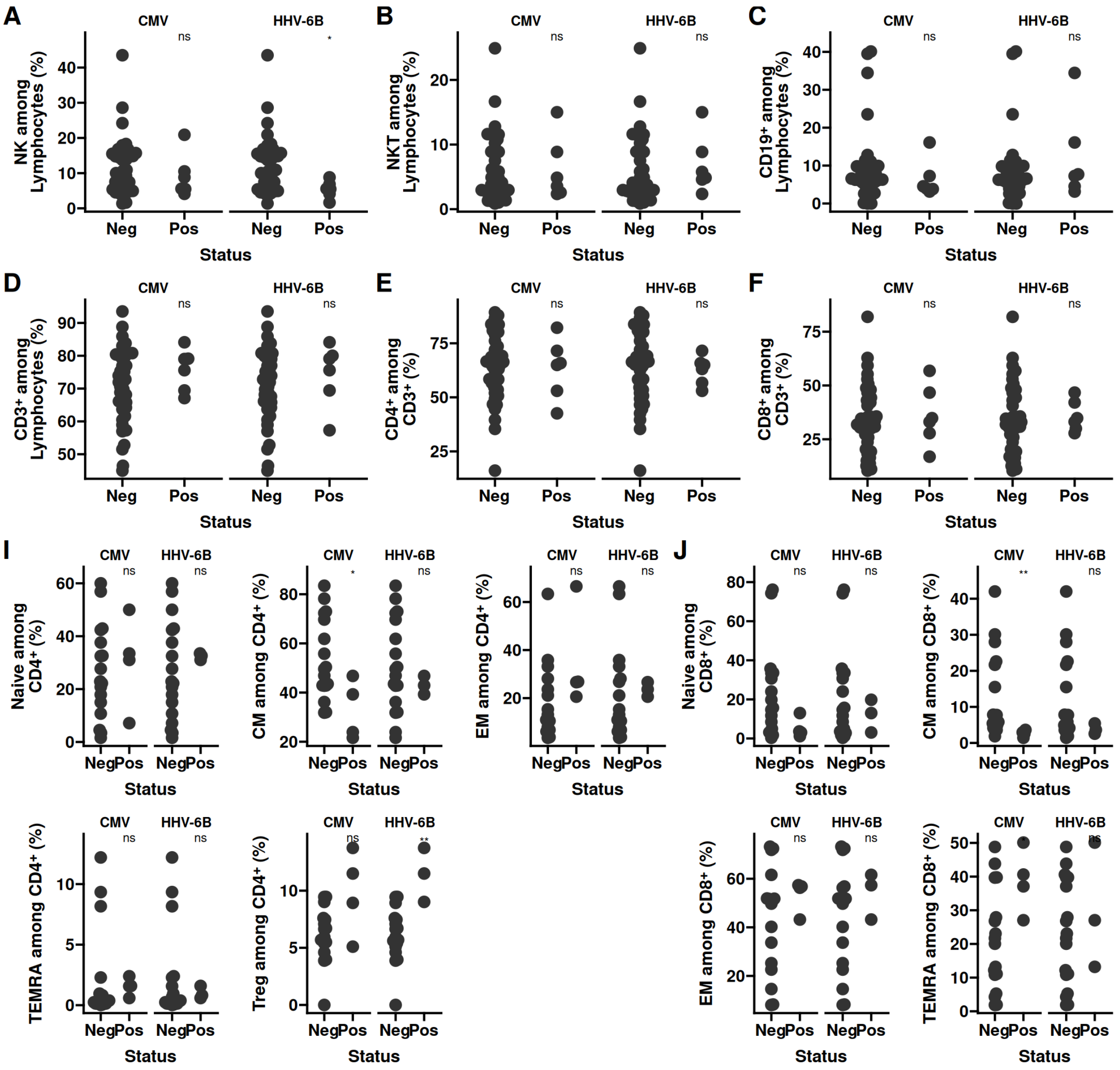

Supplementary Figure 5. Major lymphocyte populations.

Collected whole blood was evaluated for all major immune cell populations as well as memory subsets (Naive ($CD45RA^{+}CCR7^{+}$), Central memory ($CD45RA^{-}CCR7^{+}$), Effector memory ($CD45RA^{-}CCR7^{-}$), and TEMRA ($CD45RA^{+}CCR7^{-}$)) in $CD4^{+}$ and $CD8^{+}$ T cells. A) Lymphocyte count between Critical/Severe and moderate COVID-19 severity. B) Lymphocyte count in patients with and without CMV or HHV-6B reactivation. C) Frequency of NK cells in patients with and without CMV or HHV-6B reactivation. D) Frequency of NKT cells in patients with and without CMV or HHV-6B reactivation. E) Frequency of CD19 positive B cells in patients with and without CMV or HHV-6B reactivation. F) Frequency of $CD3^{+}$ T cells in patients with and without CMV or HHV-6B reactivation. G) Frequency of $CD4^{+}$ T cells in patients with and without CMV or HHV-6B reactivation. H) Frequency of $CD8^{+}$ T cells in patients with and without CMV or HHV-6B reactivation. I) Naive ($CD45RA^{+}CCR7^{+}$), Central memory ($CD45RA^{-}CCR7^{+}$), Effector memory ($CD45RA^{-}CCR7^{-}$), and TEMRA ($CD45RA^{+}CCR7^{-}$) in $CD4^{+}$ T cells. J) Naive ($CD45RA^{+}CCR7^{+}$), Central memory ($CD45RA^{-}CCR7^{+}$), Effector memory ($CD45RA^{-}CCR7^{-}$), and TEMRA ($CD45RA^{+}CCR7^{-}$) in $CD4^{+}$ T cells. CM: Central memory. EM: Effector memory. TEMRA: T Effector memory expressing CD45RA. Box plots depict the median and the first and third quartiles. The whiskers correspond to 1.5 times the interquartile range. Difference was assessed by Wilcox test.

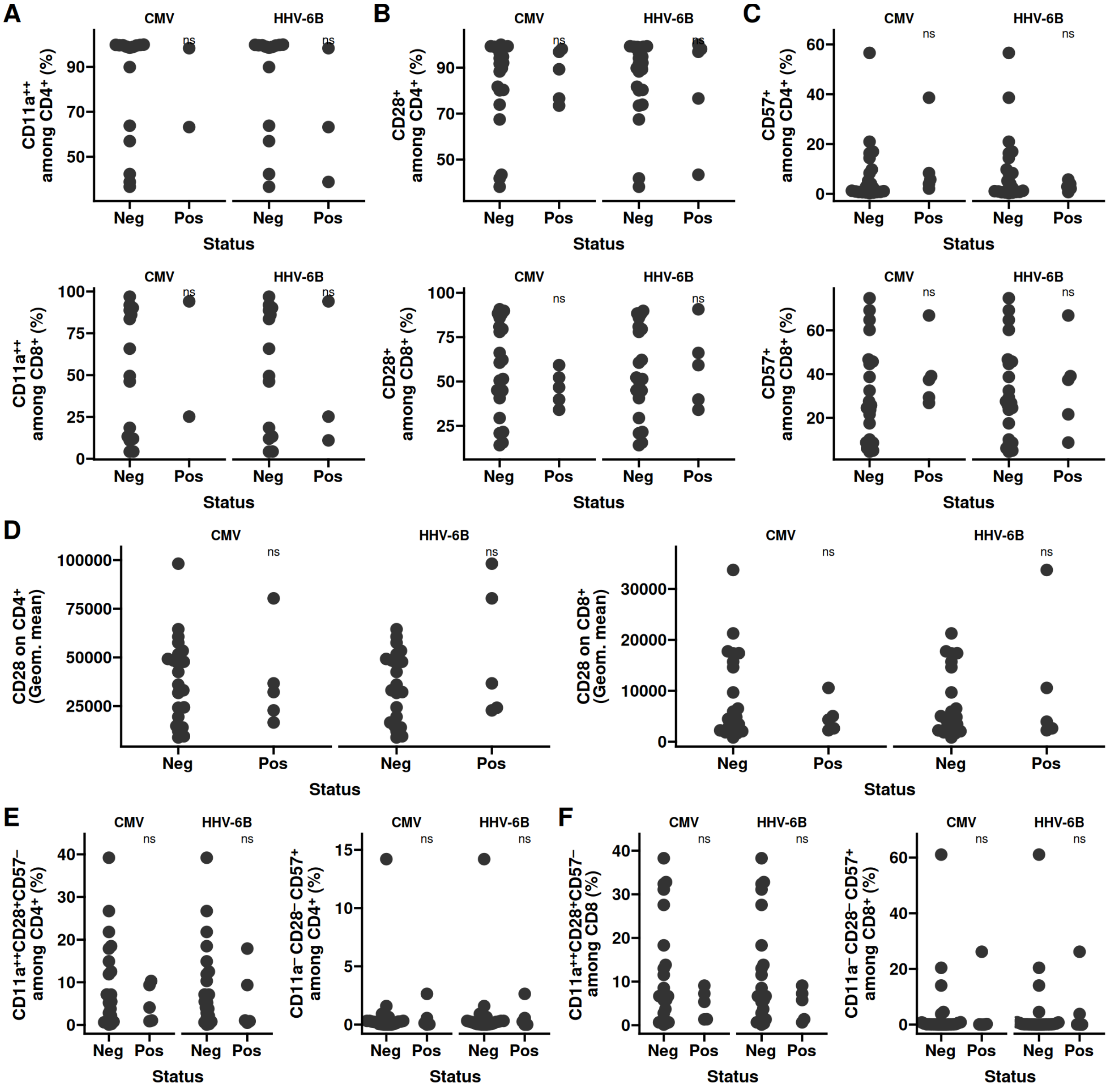

Supplementary Figure 6. T cell activation and functionality markers.

Collected whole blood was evaluated for surface expressed activation markers. A) Frequency of CD11a among CD4 T cells (top) and CD8 T cells (bottom). B) Frequency of CD28 among CD4 T cells (top) and CD8 T cells (bottom). C) Frequency of CD57 among CD4 T cells (top) and CD8 T cells (bottom). D) Geometric mean of CD28 on CD4 T cells (left) and CD8 T cells (right). E) Frequency of $CD11a^{++}CD28^{+}CD57^{-}$ (left) and $CD11a^{-}CD28^{-}CD57^{+}$ (right) on CD4 T cells. F) Frequency of $CD11a^{++}CD28^{+}CD57^{-}$ (left) and $CD11a^{-}CD28^{-}CD57^{+}$ (right) on CD8 T cells.

References

Siddiqi, Hasan K., and Mandeep R. Mehra. 2020. “COVID-19 Illness in Native and Immunosuppressed States: A Clinical-Therapeutic Staging Proposal.” *The Journal of Heart and Lung Transplantation: The Official Publication of the International Society for Heart Transplantation* 39(5):405–7. doi: 10.1016/j.healun.2020.03.012.
